# Imperatorin interacts additively with novel antiseizure medications in the mouse maximal electroshock-induced seizure model: an isobolographic transformation

**DOI:** 10.1007/s43440-023-00555-4

**Published:** 2023-11-28

**Authors:** Jarogniew J. Łuszczki, Ewelina Kochman-Moskal, Hubert Bojar, Magdalena Florek-Łuszczki, Krystyna Skalicka-Woźniak

**Affiliations:** 1 Department of Occupational Medicine, Medical University of Lublin, Lublin, Poland; 2 Department of Toxicology and Food Safety, Institute of Rural Health, Lublin, Poland; 3 Department of Medical Anthropology, Institute of Rural Health, Lublin, Poland; 4 Department of Natural Products Chemistry, Medical University of Lublin, 20-093 Lublin, Poland

**Keywords:** Antiseizure medication, Maximal electroshock-induced seizures, Pharmacodynamic interaction, Imperatorin

## Abstract

**Background:**

Anticonvulsant effects of imperatorin (IMP) have been experimentally confirmed earlier, but no information is available on the interaction profiles of this naturally occurring coumarin when combined with novel antiseizure medication (ASMs). This study aimed to determine the effects of IMP on the anticonvulsant effects of lacosamide (LCM), oxcarbazepine (OXC), pregabalin (PGB), and topiramate (TPM) in the maximal electroshock-induced seizure (MES) model in mice.

**Methods:**

The anticonvulsant effects exerted by novel ASMs (LCM, OXC, PGB, and TPM) when combined with constant doses of IMP (25 and 50 mg/kg) underwent isobolographic transformation to precisely classify the observed interactions in the mouse MES model. Total brain concentrations of ASMs were measured with high-pressure liquid chromatography to exclude the pharmacokinetic nature of interactions among IMP and the tested ASMs.

**Results:**

IMP (50 mg/kg) significantly enhanced (*p* < 0.01) the anticonvulsant potency of LCM, OXC, PGB, and TPM in the mouse MES model. IMP (25 mg/kg) mildly potentiated the anticonvulsant action of LCM, OXC, PGB, and TPM, but no statistical significance was reported for these combinations. The isobolographic transformation of data from the MES test revealed that the interactions of novel ASMs with IMP were additive. Moreover, IMP (50 mg/kg) did not affect the total brain content of any of the novel ASMs in experimental mice.

**Conclusions:**

The additive interactions of IMP with LCM, OXC, PGB, and TPM in the mouse MES model accompanied by no pharmacokinetic changes in the total brain content of ASMs are worthy of recommendation for further studies.

**Supplementary Information:**

The online version contains supplementary material available at 10.1007/s43440-023-00555-4.

## Introduction

Overwhelming evidence indicates that epilepsy is still one of the most serious and frequent neurological disorders that affects approx. 1% of the human population, which makes up around 60 million epilepsy patients worldwide [1, 2]. Despite several advances in the treatment of epilepsy, due to various novel antiseizure medications (ASMs) available for the treatment of epilepsy patients [3, 4], a proportion of treatment failure remained on a constant level affecting approx. 30% of epilepsy patients on monotherapy [5]. If monotherapy with ASM fails twice, the doctors are obliged to replace the ineffective ASMs with polytherapy containing two or more ASMs [6, 7].

For patients with epilepsy refractory to the treatment, some novel options are developed and tried by clinicians, based usually on some naturally occurring (plant-origin) compounds. The best example illustrating the introduction of a plant-origin ASM to the treatment of epilepsy is cannabidiol (Epidiolex^®^). Experimental preclinical studies indicate that one of the most intriguing substances under investigations is coumarins [8], of which imperatorin (IMP) seems the most promising agent due to its anticonvulsant effects [9], and calcium-blocking properties in preclinical studies [10, 11].

Relatively recently, it has been reported that IMP, in a dose-dependent manner, exerted the anticonvulsant action in the mouse maximal electroshock-induced seizure (MES) model [9, 12]. Additionally, IMP significantly enhanced the anticonvulsant potency of carbamazepine, phenytoin, and phenobarbital in the mouse MES model, as well as, enhanced the antiseizure action of lamotrigine in the mouse MES model [13, 14]. Of note, the mouse MES model is thought to be a model of tonic–clonic seizures and to a certain extent of partial seizures with or without secondary generalization in humans [15]. Briefly, the ASMs which are effective in suppressing tonic–clonic seizures in humans protected also the animals against tonic hindlimb extensions evoked electrically in the MES model [16].

This study was aimed at determining the effect of IMP on the anticonvulsant activity of 4 novel ASMs (namely, lacosamide (LCM), oxcarbazepine (OXC), pregabalin (PGB), and topiramate (TPM)) in the mouse maximal electroshock-induced seizure (MES) model. The rationale for investigating in this study the influence of IMP on the anticonvulsant potencies of four novel ASMs in the mouse MES model was based primarily on two premises. Firstly, novel ASMs (including, LCM, OXC, PGB, and TPM) are safer, more efficient, and better tolerated by the patients than classic ASMs [17, 18]. The studied novel ASMs (i.e., LCM, OXC, PGB, and TPM) are preferentially prescribed to patients, whose tonic–clonic seizures are not satisfactorily controlled with classic ASMs due to either intolerable high doses of ASMs used or adverse events that appear during the treatment with classic ASMs. Secondly, IMP by itself possesses anticonvulsant properties and the combination of IMP (a naturally occurring plant-derived substance) with novel ASMs could provide an efficacious antiseizure treatment with no or minimal side effects. Since the isobolographic analysis is the best method in classifying interactions between ASMs, not only in preclinical in vivo studies [19, 20], we used the subthreshold method following the isobolographic transformation to adequately and precisely verify the interaction profile for the combinations of IMP with novel ASMs in the mouse MES model. To verify the observed interactions concerning their pharmacokinetic contributions, total brain concentrations of ASMs of novel ASMs were measured with high-pressure liquid chromatography (HPLC).

## Materials and methods

### Experimental animals

Adult (8–9-week-old) male albino Swiss mice, in a total amount of 232, were used in this study. The animals were housed in a specific pathogen-free facility with a controlled environment and with free access to tap water and food (ad libitum), under standardized housing and laboratory conditions (for more details see [21]). After adaptation to laboratory conditions, the mice were randomly assigned to experimental groups comprising 8 mice per group. All efforts were made to refine procedures, protect the animals’ welfare, minimize animals’ suffering, and use only the number of animals necessary to produce reliable scientific data according to the 3Rs rule. All experimental procedures were performed in strict accordance with the ARRIVE guidelines and were approved by the Local Ethics Committee (License Nos: 88/2018 and 15/2019).

### Drugs

IMP (Sigma-Aldrich, St. Louis, MO, USA) suspended in a 1% aqueous solution of Tween 80 (Sigma-Aldrich, St. Louis, MO, USA) was administered intraperitoneally (*ip*) at 30 min. before the MES test and collection of the brain samples, as reported elsewhere [22]. LCM (Vimpat®, UCB Pharma, Brussels, Belgium), OXC (Trileptal®, Novartis Pharma AG, Basel, Switzerland), PGB (Lyrica®, Pfizer Limited, Sandwich, Kent, UK), TPM (Topamax®, Cilag AG, Schaffhausen, Switzerland) suspended in a 1% aqueous solution of Tween 80 (Sigma-Aldrich, St. Louis, MO, USA) were administered *ip* as follows: LCM and OXC-30 min, TPM-60 min and PGB-120 min before the MES test and collection of the brain samples. Of note, these pretreatment times of ASMs reflect their individual times to peak anticonvulsant effect, determined experimentally in previous studies [23, 24].

### Maximal electroshock-induced seizure (MES) test in animals

After receiving the respective doses of novel ASMs (either alone or in combination with IMP), the mice were subjected to the MES test. Electrical stimulation (50 Hz; 500 V; 25 mA; 0.2 s of duration) delivered from a generator via ear-clip electrodes evoked tonic seizure activity in all the tested mice. The protection of the mice from tonic seizure activity was expressed as median effective doses (ED_50_) of novel ASMs, according to the log-probit method [25]. To determine the ED_50_ value for each ASM, two or three experimental groups of animals were used (n = 16 or n = 24).

### Isobolographic transformation of data

Doses of IMP and the ED_50_ values of novel ASMs when used in combinations (from the MES test) were transformed to the fractions of their ED_50_ values (when used separately), as described earlier [19, 20]. The interactions between IMP and the tested drugs in the mouse MES model were characterized by the isobolographic transformation. The constant doses of IMP (25 and 50 mg/kg) in mixtures were illustrated graphically as parallel lines to the Y-axis, whereas the increasing doses of each ASM allowed creating the isoboles, as reported earlier [26].

### Measurement of total brain ASM concentrations

Pharmacokinetic estimation of total brain ASM concentrations was performed for the combinations of IMP (administered at a maximally tested dose of 50 mg/kg) with ASMs (i.e., LCM, OXC, PGB, and TPM). Thus, the measurement of total brain concentrations of LCM, OXC, PGB, and TPM was undertaken at doses that corresponded to their ED_50_ values, as determined from the MES test. After decapitation, the whole brains of mice were removed from skulls, weighed, harvested, and homogenized using Abbott buffer (1:2 w/v). After centrifugation (at 10,000 g for 10 min), the supernatant samples (200 μl) were analyzed by high-pressure liquid chromatography (HPLC) for LCM, OXC, PGB, and TPM content, as described earlier [23, 24]. Total brain ASM concentrations were expressed in μg/ml of brain supernatants as means ± SD of eight separate brain preparations.

### Statistical analysis

The ED_50_ values for novel ASMs were calculated by computer-assisted log-probit analysis [25]. The ED_50_ values (± SEM) were statistically analyzed using a one-way ANOVA test followed by Dunnett’s post-hoc test for multiple comparisons. The isobolographically transformed ED_50exp_ values (for the mixtures of IMP with each novel ASM) were statistically compared to their respective and theoretically predicted to be additive ED_50 add_ values using the unpaired Student’s t-test with Welch’s correction, as recommended elsewhere [27, 28]. Total brain ASM concentrations were statistically compared by the unpaired Student’s t-test. Differences among values were considered statistically significant if *p* < 0.05.

## Results

### Anticonvulsant effects of novel ASMs with IMP in the MES test in mice

IMP (50 mg/kg) significantly potentiated the anticonvulsant action of LCM, OXC, PGB, and TPM in the mouse MES model (**p < 0.01; Table 1). On the contrary, IMP (25 mg/kg) had no significant impact on the anticonvulsant potencies of all the tested novel ASMs (LCM, OXC, PGB, and TPM) in the mouse MES model, albeit a slight reduction in the ED_50_ values of the novel ASMs was reported (Table 1).

**Table 1:** Influence of IMP on the anticonvulsant effects of novel ASMs in the maximal electroshock (MES)-induced seizure model in mice

Drug combination	ED50 (± SEM)	n	one-way ANOVA
LCM + vehicle	8.27 ± 1.20	16	
LCM + IMP (25)	6.52 ± 0.88	16	
LCM + IMP (50)	3.38 ± 0.93**	16	F_2,45_ = 5.981, *p* = 0.005
OXC + vehicle	9.72 ± 1.07	24	
OXC + IMP (25)	6.62 ± 0.96	16	
OXC + IMP (50)	4.80 ± 0.81**	16	F_2,53_ = 6.477, *p* = 0.003
PGB + vehicle	197.6 ± 12.6	24	
PGB + IMP (50)	165.0 ± 12.1	24	
PGB + IMP (50)	134.6 ± 11.8**	16	F_2,61_ = 5.877, *p* = 0.0046
TPM + vehicle	90.05 ± 10.54	24	
TPM + IMP (25)	74.84 ± 7.52	16	
TPM + IMP (50)	54.14 ± 6.09**	24	F_2,61_ = 4.979, *p* = 0.0099

Data are presented as median effective doses (ED_50_ in mg/kg ± SEM) of four novel ASMs when administered alone and combined with IMP (in doses of 25 or 50 mg/kg) in the maximal electroshock (MES)-induced seizure model. The ED_50_ values were calculated from the computer-assisted log-probit method

*n* number of animals at those doses, for which the anticonvulsant effects ranged between the 4th and 6th probit

**p < 0.01 vs the respective control (vehicle-treated) animals (one-way ANOVA followed by the post-hoc Dunnett’s test). Total number of animals used was 232

### Isobolographic transformation of interactions between IMP and novel ASMs in the MES test in mice

**Fig. 1:**
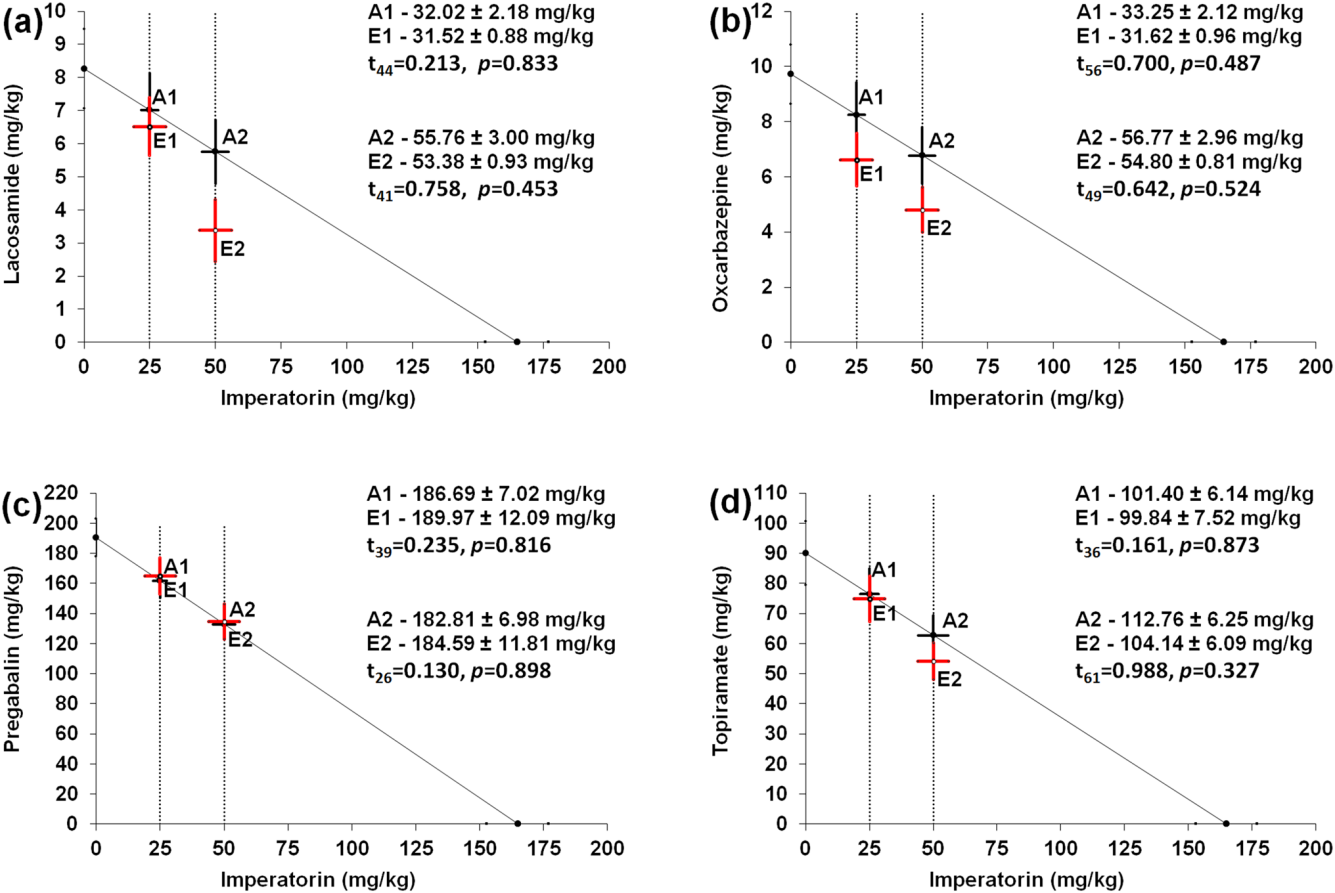
**A**–**D** Isobolograms with additive interactions between IMP and LCM (**A**), OXC (**B**), PGB (**C**), and TPM (**D**) in the MES-induced seizure model in mice. The ED_50_ values for IMP and four novel ASMs were plotted graphically on the X- and Y-axis, respectively. The line segments on both axes represent SEM values for the ED_50_ values. The dotted lines collateral to the Y-axis reflect the constant doses of IMP (25 and 50 mg/kg). The points A1 and A2 depict the theoretical additive ED_50 add_ values. Points E1 and E2 represent the experimentally derived ED_50 mix_ values for the mixtures of IMP with one of the tested novel ASM. The unpaired Student’s t-test with Welch’s correction revealed no significance between the investigated ED_50 add_ and ED_50 mix_ values, indicating additive interactions between IMP and novel ASMs. Total number of animals used was 232

Isobolographic comparison of the respective ED_50 exp_ with ED_50 add_ values (for each combination separately) revealed no significant differences between these values in the MES test in mice, confirming the additive nature of interactions between the tested drugs (Fig. 1A–D).

### Influence of IMP on total brain concentrations of novel ASMs

The total brain content of LCM, OXC, PGB, and TPM remained unchanged in experimental animals when IMP (50 mg/kg) was added to novel ASMs (Table 2).

**Table 2:** Influence of IMP on total brain concentrations of ASMs in mice

Drug combination	ASM (µg/ml)	n	Student’s t-test
LCM (3.38) + vehicle	0.252 ± 0.011	8	
LCM (3.38) + IMP (50)	0.248 ± 0.015	8	t_14_ = 0.608, *p* = 0.553
OXC (4.80) + vehicle	0.921 ± 0.052	8	
OXC (4.80) + IMP (50)	0.937 ± 0.061	8	t_14_ = 0.565, *p* = 0.581
PGB (134.6) + vehicle	64.87 ± 2.51	8	
PGB (134.6) + IMP (50)	65.19 ± 2.62	8	t_14_ = 0.250, *p* = 0.807
TPM (54.14) + vehicle	6.861 ± 0.442	8	
TPM (54.14) + IMP (50)	7.014 ± 0.483	8	t_14_ = 0.661, *p* = 0.519

Data are presented as mean concentrations (in µg/ml ± SD) of ASMs in the brain tissue of experimental animals

*n* number of animals’ brain tissue used

## Discussion

IMP (50 mg/kg) significantly potentiated the anticonvulsant action of all the studied novel ASMs (LCM, OXC, PGB, and TPM) in the mouse MES model. The enhancing effects of IMP on the anticonvulsant action of LCM, OXC, PGB, and TPM are in line with results reported earlier for classic ASMs (i.e., CBZ, PB, and PHT) and LTG in the mouse MES model [13, 14]. Due to the isobolographic transformation of data, used to correctly classify the interactions between IMP and novel ASMs, it was found that the combinations of IMP with novel ASMs exerted additive interactions in the mouse MES model. Although IMP potentiated the antiseizure effects of some novel ASMs, the isobolography revealed only additive interactions proving that only this method can correctly classify the interactions observed in preclinical conditions. The superiority of isobolographic analysis over the subthreshold method used in experimental epileptology has been previously confirmed [26, 29]. Considering the anticonvulsant potential of all naturally-occurring coumarins tested, it should be stated that IMP possesses the best anticonvulsant profile potentiating the effects of several ASMs in the mouse MES model, in contrast to osthole, umbelliferone, xanthotoxin, and scoparone (Supplementary Table 1).

Previously, it has been documented that isopimpinellin (ISOP—another naturally-occurring coumarin) exerted additive interactions when combined with CBZ, PB, and PHT, and simultaneously, it produced antagonistic interaction for the combination with VPA in the mouse MES model [30]. Of note, any antagonistic interaction in terms of seizure suppression is unfavorable, from a pharmacological viewpoint, due to the decrease in the anticonvulsant potential of the drugs in a mixture [31, 32]. In the case of scoparone, this naturally occurring coumarin exerted additive interactions with CBZ, PHT, PB, and VPA in the mouse MES model [26]. Unfortunately, the anticonvulsant effects for the combinations of classic ASMs with osthole, umbelliferone, and xanthotoxin have not been isobolographically transformed and the exact types of interactions for these drug combinations are unknown as yet.

From a theoretical viewpoint, the additive interaction between two drugs may be clinically efficacious because of the low doses of both drugs administered to the patients offering the same anticonvulsant effects. If monotherapy with ASM is conducted in maximally tolerated doses of an ASM, some adverse effects may occur [33–35]. In such a situation, a duo-therapy with low doses of both ASMs may be helpful for epilepsy patients offering seizure suppression with concomitant reduction of adverse effects accompanied by the treatment with ASM in monotherapy [6, 36]. The combinations of ASMs exerting additive interactions can be preferentially chosen by clinicians to reduce adverse effects associated with high-dose ASM treatment. Hence, the combinations of IMP with novel ASMs offering the additive interactions in the mouse MES model deserve in future clinical attention.

In this study, the total brain concentrations of ASMs were estimated to exclude any pharmacokinetic interactions between the tested ASMs and IMP. As reported earlier, IMP significantly elevated the total brain content of CBZ, but not that of PHT, PB, and VPA in the mice [13]. In this study, none of the tested ASMs (i.e., LCM, OXC, PGB, and TPM) significantly changed their concentrations after IMP administration. With HPLC we confirmed that the observed interactions between IMP and novel ASMs were pharmacodynamic. Of note, only total brain concentrations of ASMs adequately and precisely characterize the interactions. As reported earlier, plasma concentrations of ASMs may sometimes differ considerably from the total brain concentrations in experimental animals (for more details see: [37, 38]). The brain concentrations of ASMs adequately illustrate the interactions of drugs in the site(s) of action of ASMs, where the drugs produce their anticonvulsant action.

It is important to note that in this study we did not determine the influence of IMP on the acute adverse effects produced by the novel ASMs in mice challenged with 3 standard behavioral tests (namely, chimney test, step-through passive avoidance task, and grip strength test). Previously, we have reported no significant deficits in retention times in the step-through passive avoidance task in mice who received the combinations of IMP with classic ASMs [13]. Additionally, neither impairment of motor coordination, nor skeletal muscular strength changes were documented in mice challenged with the chimney and grip-strength tests, respectively [13]. Since the acute neurotoxic profile of some novel ASMs (LCM, OXC, PGB, and TPM) is better than classic ASMs in preclinical studies [32, 39–41], it was not necessary to perform additional experiments on animals to confirm that the drugs in doses from the MES test would be devoid of any acute adverse effects. Another premise in order not to conduct additional experiments on animals in the chimney, step-through passive avoidance and grip-strength tests in mice, linked with the “3R rule” (Reduction, Replacement, Refinement) in animal in vivo studies [42, 43], was taken into consideration when constructing the research protocols without assessment of motor coordination, long-term memory and skeletal muscular strength in animals.

The principal limitation in this study is linked with the assessment of interaction profiles for the combinations between ASMs and IMP after a single (acute) administration of the drugs. Since no chronic treatment experiments were performed when determining the interaction profiles of IMP in combinations with LCM, OXC, PGB, and TPM, some pharmacokinetic changes associated with chronic (long-term) drugs’ administration, distribution, metabolism, and elimination might significantly affect the anticonvulsant effects observed in mice. On the other hand, due to some fundamental interspecies differences between mice and humans, the results from chronic experiments on mice must not be directly transferred to clinical conditions. Despite the above-mentioned limitations, this in vivo study provides us with information about the anticonvulsant activity of IMP when administered in combinations with novel ASMs that would be potentially useful after further preclinical verification.

## Conclusions

The isobolographically determined additive interactions of IMP with LCM, OXC, PGB, and TPM in the model of tonic–clonic seizures in mice deserve recommendations for further studies because of the lack of changes in total brain concentrations of ASMs after IMP administration.

### Supplementary Information

Below is the link to the electronic supplementary material.

Supplementary file1 (DOC 28 KB)

## Data Availability

The datasets generated during and/or analyzed during the current study are available from the corresponding author upon reasonable request.

## Abbreviations

ASMs: Antiseizure medications
IMP: Imperatorin
LCM: Lacosamide
MES: Maximal electroshock-induced seizure model
OXC: Oxcarbazepine
PGB: Pregabalin
TPM: Topiramate

## References

[1] Bialer M, Johannessen SI, Koepp MJ, Levy RH, Perucca E, Tomson T, White HS (2018). Progress report on new antiepileptic drugs: a summary of the fourteenth eilat conference on new antiepileptic drugs and devices (EILAT XIV). I. Drugs in preclinical and early clinical development. Epilepsia.

[2] Bialer M, Johannessen SI, Koepp MJ, Levy RH, Perucca E, Tomson T, White HS (2018). Progress report on new antiepileptic drugs: a summary of the fourteenth eilat conference on new antiepileptic drugs and devices (EILAT XIV). II. Drugs in more advanced clinical development. Epilepsia.

[3] Bialer M, Johannessen SI, Koepp MJ, Levy RH, Perucca E, Perucca P (2022). Progress report on new antiepileptic drugs: a summary of the sixteenth eilat conference on new antiepileptic drugs and devices (EILAT XVI): II. Drugs in more advanced clinical development. Epilepsia.

[4] Bialer M, Johannessen SI, Koepp MJ, Levy RH, Perucca E, Perucca P (2022). Progress report on new antiepileptic drugs: a summary of the sixteenth eilat conerence on new antiepileptic drugs and devices (EILAT XVI): I. Drugs in preclinical and early clinical development. Epilepsia.

[5] Janmohamed M, Brodie MJ, Kwan P (2020). Pharmacoresistance-epidemiology, mechanisms, and impact on epilepsy treatment. Neuropharmacology.

[6] Stephen LJ, Brodie MJ (2012). Antiepileptic drug monotherapy versus polytherapy: pursuing seizure freedom and tolerability in adults. Curr Opinion Neurol.

[7] Graham-Rowe E, Katzer CB, Riaz S, Attwood A, Bates L, Sainz-Fuertes R, Swan B (2023). Unmet needs of people with epilepsy: a qualitative study exploring their journey from presentation to long-term management across five European countries. Front Neurol.

[8] Challal S, Skiba A, Langlois M, Esguerra CV, Wolfender JL, Crawford AD, Skalicka-Woźniak K (2023). Natural product-derived therapies for treating drug-resistant epilepsies: from ethnopharmacology to evidence-based medicine. J Ethnopharmacol.

[9] Luszczki JJ, Glowniak K, Czuczwar SJ (2007). Time-course and dose-response relationships of imperatorin in the mouse maximal electroshock seizure threshold model. Neurosci Res.

[10] Lu W, Zhang T, Li P, Wang F, Pan X, Wang C (2013). Structural modeling and identification of imperatorin as novel L-type calcium channel blocker. Med Chem.

[11] Lee E, Choi SY, Yang JH, Lee YJ (2016). Preventive effects of imperatorin on perfluorohexanesulfonate-induced neuronal apoptosis via inhibition of intracellular calcium-mediated ERK pathway. Korean J Physiol Pharmacol.

[12] Luszczki JJ, Andres-Mach M, Glensk M, Skalicka-Wozniak K (2010). Anticonvulsant effects of four linear furanocoumarins, bergapten, imperatorin, oxypeucedanin, and xanthotoxin, in the mouse maximal electroshock-induced seizure model: a comparative study. Pharmacol Rep.

[13] Luszczki JJ, Glowniak K, Czuczwar SJ (2007). Imperatorin enhances the protective activity of conventional antiepileptic drugs against maximal electroshock-induced seizures in mice. Eur J Pharmacol.

[14] Luszczki JJ, Wojda E, Raszewski G, Glowniak K, Czuczwar SJ (2008). Influence of imperatorin on the anticonvulsant activity and acute adverse-effect profile of lamotrigine in maximal electroshock-induced seizures and chimney test in mice. Pharmacol Rep.

[15] Löscher W (2017). Animal Models of Seizures and Epilepsy: Past, Present, and Future Role for the Discovery of Antiseizure Drugs. Neurochem Res.

[16] Löscher W, Fassbender CP, Nolting B (1991). The role of technical, biological, and pharmacological factors in the laboratory evaluation of anticonvulsant drugs. II. Maximal electroshock seizure models. Epilepsy Res.

[17] Billakota S, Devinsky O, Kim KW (2020). Why we urgently need improved epilepsy therapies for adult patients. Neuropharmacology.

[18] Perucca E (2019). Antiepileptic drugs: evolution of our knowledge and changes in drug trials. Epileptic Disord.

[19] Luszczki JJ, Danysz W, Czuczwar SJ (2008). Interactions of MRZ 2/576 with felbamate, lamotrigine, oxcarbazepine and topiramate in the mouse maximal electroshock-induced seizure model. Pharmacology.

[20] Luszczki JJ, Marzeda P, Gut-Lepiech A, Kondrat-Wrobel MW, Wroblewska-Luczka P, Karwan S, Plech T (2019). New derivative of 1,2,4-triazole-3-thione (TP427) potentiates the anticonvulsant action of valproate, but not that of carbamazepine, phenytoin or phenobarbital in the mouse tonic-clonic seizure model. Pharmacol Rep.

[21] Luszczki JJ, Panasiuk A, Zagaja M, Karwan S, Bojar H, Plewa Z, Florek-Łuszczki M (2020). Polygonogram and isobolographic analysis of interactions between various novel antiepileptic drugs in the 6-Hz corneal stimulation-induced seizure model in mice. PLoS ONE.

[22] Luszczki JJ, Wojda E, Andres-Mach M, Cisowski W, Glensk M, Glowniak K, Czuczwar SJ (2009). Anticonvulsant and acute neurotoxic effects of imperatorin, osthole and valproate in the maximal electroshock seizure and chimney tests in mice: a comparative study. Epilepsy Res.

[23] Luszczki JJ, Wlaz A, Karwan S, Florek-Luszczki M, Czuczwar SJ (2013). Effects of WIN 55,212–2 mesylate on the anticonvulsant action of lamotrigine, oxcarbazepine, pregabalin and topiramate against maximal electroshock-induced seizures in mice. Eur J Pharmacol.

[24] Luszczki JJ, Zagaja M, Miziak B, Kondrat-Wrobel MW, Zaluska K, Wroblewska-Luczka P (2018). Beneficial combination of lacosamide with retigabine in experimental animals: an isobolographic analysis. Pharmacology.

[25] Litchfield JT, Wilcoxon F (1949). A simplified method of evaluating dose-effect experiments. J Pharmacol Exp Ther.

[26] Łuszczki JJ, Bojar H, Góralczyk A, Skalicka-Woźniak K (2023). Antiseizure effects of scoparone, borneol and their impact on the anticonvulsant potency of four classic antiseizure medications in the mouse MES model-an isobolographic transformation. Int J Mol Sci.

[27] Tallarida RJ (2006). An overview of drug combination analysis with isobolograms. J Pharmacol Exp Ther.

[28] Tallarida RJ (2016). Drug combinations: tests and analysis with isoboles. Curr Protocols Pharmacol.

[29] Luszczki JJ, Czuczwar SJ (2003). Isobolographic and subthreshold methods in the detection of interactions between oxcarbazepine and conventional antiepileptics–a comparative study. Epilepsy Res.

[30] Luszczki JJ, Bojar H, Jankiewicz K, Florek-Łuszczki M, Chmielewski J, Skalicka-Woźniak K (2023). Anticonvulsant effects of isopimpinellin and its interactions with classic antiseizure medications and borneol in the mouse tonic-clonic seizure model: an isobolographic transformation. Pharmacol Rep.

[31] Tallarida RJ (2007). Interactions between drugs and occupied receptors. Pharmacol Ther.

[32] Luszczki JJ, Czuczwar SJ (2004). Preclinical profile of combinations of some second-generation antiepileptic drugs: an isobolographic analysis. Epilepsia.

[33] Tomson T, Zelano J, Dang YL, Perucca P (2023). The pharmacological treatment of epilepsy in adults. Epileptic Disord.

[34] Perucca E, Perucca P, White HS, Wirrell EC (2023). Drug resistance in epilepsy. Lancet Neurol.

[35] Marques VD, Hackbart BA, Guilhoto LM, Duarte JTC, Peixoto-Santos JE, Yacubian EMT, Bittar Guaranha MS (2023). Minimum effective sodium valproate dose in genetic generalized epilepsies. Seizure.

[36] Stephen LJ, Forsyth M, Kelly K, Brodie MJ (2012). Antiepileptic drug combinations–have newer agents altered clinical outcomes?. Epilepsy Res.

[37] Luszczki JJ, Ratnaraj N, Patsalos PN, Czuczwar SJ (2005). Pharmacodynamic and/or pharmacokinetic characteristics of interactions between loreclezole and four conventional antiepileptic drugs in pentylenetetrazole-induced seizures in mice: an isobolographic analysis. Epilepsy Behav.

[38] Luszczki JJ, Ratnaraj N, Patsalos PN, Czuczwar SJ (2006). Isobolographic analysis of interactions between loreclezole and conventional antiepileptic drugs in the mouse maximal electroshock-induced seizure model. Naunyn Schmiedebergs Arch Pharmacol.

[39] Zadrozniak A, Wojda E, Wlaz A, Luszczki JJ (2009). Characterization of acute adverse-effect profiles of selected antiepileptic drugs in the grip-strength test in mice. Pharmacol Rep.

[40] Stohr T, Kupferberg HJ, Stables JP, Choi D, Harris RH, Kohn H (2007). Lacosamide, a novel anti-convulsant drug, shows efficacy with a wide safety margin in rodent models for epilepsy. Epilepsy Res.

[41] Łuszczki JJ, Zadrożniak A, Wlaź A, Andres-Mach M, Dudra-Jastrzębska M, Zwoliński J (2008). Characterization of acute adverse-effect profi le of carbamazepine and valproate in the grip-strength test in mice. J Pre-Clin Clin Res.

[42] Hubrecht RC, Carter E (2019). The 3Rs and humane experimental technique: implementing change. Animals.

[43] Kilkenny C, Browne W, Cuthill IC, Emerson M, Altman DG (2010). Animal research: reporting in vivo experiments: the ARRIVE guidelines. Brit J Pharmacol.

