# Supplementary material for: Imperatorin interacts additively with novel antiseizure medications in the mouse maximal electroshock-induced seizure model: an isobolographic transformation
Source: Pharmacol Rep. 2023 Nov 28;76(1):216–22. doi: 10.1007/s43440-023-00555-4 (PMC10830790; doi:10.1007/s43440-023-00555-4)
Supplement: Supplementary file 1 — Supplementary file1 (DOC 28 KB) [file 43440_2023_555_MOESM1_ESM.doc]

**Supplementary Table 1.** Influence of selected naturally-occurring coumarins on the anticonvulsant potency of classic ASMs in the mouse MES model.

-----------------------------------------------------------------------------------------------------

Drugs CBZ PHT PB VPA Reference

-----------------------------------------------------------------------------------------------------

IMP ↑ ↑ ↑ 0 [1]

OST 0 0 0 0 [2, 3]

UMB 0 0 ↑ ↑ [4]

XNT ↑ 0 0 ↑ [5]

SCP 0 0 ↑ ↑ [6]

ISOP 0 0 0 0 [7]

-----------------------------------------------------------------------------------------------------

CBZ – carbamazepine; PHT – phenytoin; PB – phenobarbital; VPA – valproate; IMP – imperatorin; OST – osthole; UMB – umbelliferone; XNT – xanthotoxin; SCP – scoparone; ISOP – isopimpinellin; ↑ - enhancement of the anticonvulsant effect; 0 – no significant effect.

References:

[1] Luszczki JJ, Glowniak K, Czuczwar SJ. Imperatorin enhances the protective activity of conventional antiepileptic drugs against maximal electroshock-induced seizures in mice. Eur J Pharmacol. 2007;574:133-139.

[2] Luszczki JJ, Rekas A, Mazurkiewicz LR, Gleńsk M, Ossowska G. Effect of osthole on the protective activity of carbamazepine and phenobarbital against maximal electroshock-induced seizures in mice. Ann Univ Mariae Curie-Sklodowska, Sect DDD Pharm. 2010;23:145–156.

[3] Luszczki JJ, Marczewski T, Mazurkiewicz LP, Karwan S, Teresińska M, Florek-Łuszczki M. Influence of osthole on the anticonvulsant activity of phenytoin and valproate in the maximal electroshock-induced seizures in mice. Ann Univ Mariae Curie-Sklodowska, Sect DDD Pharm 2011;24:33-44.

[4] Zagaja M, Andres-Mach M, Skalicka-Wozniak K, Rekas AR, Kondrat-Wrobel MW, Glensk M, Luszczki JJ. Assessment of the combined treatment with umbelliferone and four classical antiepileptic drugs against maximal electroshock-induced seizures in mice. Pharmacology. 2015;96:175-180.

[5] Zagaja M, Pyrka D, Skalicka-Wozniak K, Glowniak K, Florek-Luszczki M, Glensk M, Luszczki JJ. Effect of xanthotoxin (8-methoxypsoralen) on the anticonvulsant activity of classical antiepileptic drugs against maximal electroshock-induced seizures in mice. Fitoterapia. 2015;105:1-6.

[6] Luszczki JJ, Bojar H, Góralczyk A, Skalicka-Woźniak K. Antiseizure Effects of Scoparone, Borneol and Their Impact on the Anticonvulsant Potency of Four Classic Antiseizure Medications in the Mouse MES Model-An Isobolographic Transformation. Int. J Mol. Sci. 2023;24(2):1395.

[7] Luszczki JJ, Bojar H, Jankiewicz K, Florek-Łuszczki M, Chmielewski J, Skalicka-Woźniak K, Anticonvulsant effects of isopimpinellin and its interactions with classic antiseizure medications and borneol in the mouse tonic-clonic seizure model: an isobolographic transformation. Pharmacol Rep. 2023; doi: 10.1007/s43440-023-00532-x.
